# N-acetylcysteine decreases malignant characteristics of glioblastoma cells by inhibiting Notch2 signaling

**DOI:** 10.1186/s13046-018-1016-8

**Published:** 2019-01-03

**Authors:** Jie Deng, An-Dong Liu, Guo-Qing Hou, Xi Zhang, Kun Ren, Xuan-Zuo Chen, Shawn S. C. Li, Yao-Song Wu, Xuan Cao

**Affiliations:** 1 School of Basic Medicine, Tongji Medical College, Huazhong University of Science and Technology, Wuhan, 430030 China; 2 School of Basic Medical Sciences, Wenzhou Medical University, Wenzhou, China; 3 Department of Biochemistry, Schulich School of Medicine and Dentistry, Western University, London, Ontario Canada; 4 The Institute of Cancer Molecular Mechanisms & Drug Targets, School of Basic Medicine, Henan University of Traditional Chinese Medicine, Zhengzhou, China; 5 Shenzhen Huazhong University of Science and Technology Research Institute, Shenzhen, China

**Keywords:** Glioblastoma, Notch2, N-acetylcysteine, Buthionine sulfoximine, Lysosome, Itch

## Abstract

**Background:**

Glioblastomas multiforme (GBM) is the most devastating primary intracranial malignancy lacking effective clinical treatments. Notch2 has been established to be a prognostic marker and probably involved in GBM malignant progression. N-acetylcysteine (NAC), a precursor of intracellular glutathione (GSH), has been widely implicated in prevention and therapy of several cancers. However, the role of NAC in GBM remains unclear and the property of NAC independent of its antioxidation is largely unknown.

**Methods:**

The mRNA and protein levels of Notch family and other related factors were detected by RT-PCR and western blot, respectively. In addition, intracellular reactive oxygen species (ROS) was measured by flow cytometry-based DCFH-DA. Moreover, cell viability was assessed by CCK8 and cell cycle was analyzed by flow cytometry-based PI staining. The level of apoptosis was checked by flow cytometry-based Annexin V/PI. Cell migration and invasion were evaluated by wound healing and transwell invasion assays. At last, U87 Xenograft model was established to confirm whether NAC could restrain the growth of tumor.

**Results:**

Our data showed that NAC could decrease the protein level of Notch2. Meanwhile, NAC had a decreasing effect on the mRNA and protein levels of its downstream targets Hes1 and Hey1. These effects caused by NAC were independent of cellular GSH and ROS levels. The mechanism of NAC-mediated Notch2 reduction was elucidated by promoting Notch2 degradation through Itch-dependent lysosome pathway. Furthermore, NAC could prevent proliferation, migration, and invasion and might induce apoptosis in GBM cells via targeting Notch2. Significantly, NAC could suppress the growth of tumor in vivo.

**Conclusions:**

NAC could facilitate Notch2 degradation through lysosomal pathway in an antioxidant-independent manner, thus attenuating Notch2 malignant signaling in GBM cells. The remarkable ability of NAC to inhibit cancer cell proliferation and tumor growth may implicate a novel application of NAC on GBM therapy.

**Electronic supplementary material:**

The online version of this article (10.1186/s13046-018-1016-8) contains supplementary material, which is available to authorized users.

## Introduction

Glioblastomas multiforme (GBM) is the most malignant brain tumor which is characterized by rapid proliferation, aggressive infiltration and early recurrence during its progression [1, 2]. Multiple signaling pathways are involved in the development of GBM, among which Notch is reported frequently and has an important impact on GBM cell growth [3].

The Notch proteins (Notch 1–4) are evolutionarily conserved transmembrane receptors which control key steps of development, cell growth and differentiation [4]. The activation of Notch signaling is initiated by its ligands (Jagged 1, 2, Delta-like 1, 3, 4) on an adjacent cell and subsequently triggers 2 successive cleavages-mediated proteolytic release of the Notch intracellular domain (NICD) [5]. NICD then translocates into the nucleus where it could bind to CBF1/Su (H)/LAG1(CSL) and recruits other coactivators to trigger the transcriptional activation of the downstream targets such as Hes1 and Hey1 [6]. Dysregulated Notch signaling has been implicated in the genesis of many human cancers including GBM [7, 8]. Targeting Notch signaling by N-[N-(3, 5-difluorophenacetyl)-L-alanyl]-S-phenylglycine t-butyl ester (DAPT), a gamma-secretase inhibitor, can suppress GBM progression via uncoupling of tumor vessel density from vessel function [9, 10]. It has been demonstrated that aberrant expression of Notch2 may play a role in gliomagenesis and Notch2 can serve as a negative predictor of survival in human glial brain tumors [11, 12]. Knockdown of Notch receptors individually revealed that Notch1 and Notch2 contributed to GBM cell growth, of which Notch2 could play a predominant role [7].

N-acetylcysteine (NAC), a precursor of reduced glutathione (GSH), has been widely used as an antioxidant against reactive oxygen species (ROS) in several disorders related to oxidative stress [8], in addition to its applications in ischemia–reperfusion injury, acute respiratory distress syndrome and chemotherapy-induced toxicity [13–15], NAC has also been proposed as an anticancer agent in vitro and in vivo either stand-alone or as an adjuvant to reduce aggressiveness in several cancers [16, 17]. Although NAC is best known as its antioxidant activity, it has been reported that its usual mechanism of increasing intracellular GSH is not required for NAC-induced G1 arrest in papilloma cells [18]. NAC can cause G1 arrest via MAPK pathway in hepatic stellate cells (HSC), which is also independent of intracellular GSH level [9]. Since accumulating evidences support that other molecular mechanisms mediating the non-antioxidant effect of NAC may exist, it is desperately needed to take a further investigation on the mechanism underlying the effect of NAC.

Here we have shown that NAC could effectively suppress Notch2 and its downstream signaling which would prevent the malignancy of GBM through GSH-independent and lysosome-mediated pathways. These findings may have implications for a new application of NAC on GBM therapy.

## Materials and methods

### Cell culture

U87 cell line was obtained from American Tissue Culture Collection (Manassas, VA, USA). U251 cell line was obtained from the Cell Bank of Chinese Academy of Sciences (Shanghai, China). Both cell lines were cultured in DMEM (Invitrogen, Carlsbad, VA, USA) supplemented with 10% fetal bovine serum (Gibco), 1% penicillin/streptomycin (Solarbio, Beijing, China) and 2% L-glutamine (Mediatech, Manassas, VA, USA). Cells were maintained in a humidified incubator at 37 °C with 5% CO2.

### Plasmid construction and transient transfection

The ORF of the Notch2 cDNA was amplified by RT-PCR using specific primers (forward, 5′-ATG CCC GCC CTG CGC CCC GCT CT-3′ and reverse, 5′-TTA TAA CTT AAG ACA ATG CCC T-3′) that were designed based on the Notch2 gene (GenBank ID: NM_001200001.1) by Takara (Shiga, Japan). The gel-purified PCR products were digested with the restriction enzymes, EcoR I and Not I (New England Biolabs), and cloned into the pcDNA3.1 vector (Invitrogen). The inserted sequence was confirmed by DNA sequencing. Scramble, Notch2 siRNA (5’-GUG CCA GAC AGA CAU GAA UTT-3′), Notch3 siRNA (5’-CCU GGC UAC AAU GGU GAU ATT-3′) and Itch siRNA (5′-AAG UGC UUC UCA GAA UGA UGA-3′) were purchased from GenePharma (Shanghai, China). The pcDNA3.1 vector (EV, empty vector) and pcDNA3.1 Notch2 was electroporated into cells following the reported program of Lonza® Nucleofector® II electroporation system. SiRNA transfections were carried out using Lipofectamine 3000 (Invitrogen) following the manufacturer’s instructions. After 36 h of transfection, the cells were harvested and analyzed for the expression.

### Western blot

Cells were collected and mixed with lysis buffer (Beyotime, Shanghai, China) containing 1 mM phenylmethylsulfonyl fluoride (PMSF) (Beyotime) for lysis at 4 °C for 30 min. Next, the mixture was centrifuged and the supernatant was used to determine the protein concentration with a BCA kit (Beyotime), and protein samples were then mixed with 5 × sodium dodecyl sulfate (SDS) loading buffer (Beyotime) prior to denaturation in the boiling water bath for 5 min. Subsequently, the samples were resolved by 6–12% SDS-PAGE, transferred to a PVDF membrane (Millipore, Billerica, MA, USA), and then blocked with 5% milk in TBST at room temperature for 1 h. The membrane was then incubated with Notch1(Santa Cruz Biotechnology, CA, USA, sc-376,403), Notch2(Cell Signaling Technology, Danvers, MA, USA, 4530), Notch3(Santa, sc-5593), Notch4(R&D, MAB3847), Hes1(Millipore, NG1839542), Hey1(Millipore, NG1829781), Itch(Santa, sc-28,367), CRMP5(Santa, sc-58,515), P21(Abcam, Cambridge, UK, England, ab109520), CDK2(Abcam, ab32147), Cyclin E (Abcam, ab33911), Bax(Cell Signaling Technology, 5023), Bcl-2(Cell Signaling Technology, 15,071) or β-actin(Proteintech, Wuhan, China, 60,008–1-lg) respectively at 4 °C overnight and followed by a secondary anti-rabbit or anti-mouse antibody (Cell Signaling Technology) at room temperature for 1 h. The membrane was detected with an enhanced chemiluminescence detection kit (Pierce, Thermo Scientific, USA). Loading was normalized with β-actin.

### Real-time PCR

As previously described [19], the total RNA was isolated by the Trizol method (Invitrogen) and reversely transcripted to cDNA with FastKing RT Kit (TIANGEN, Beijing, China). Real-time PCR analyses of mRNA levels were performed with THUNDERBIRD® SYBR® qPCR Mix (TOYOBO, Japan). The forward and reverse primer pairs were as follows: Notch2, 5′-TCA ACT GCC AAG CGG ATG T-3′ and 5′-CTT GGC TGC TTC ATA GCT CC-3′; Hes1, 5′-GTC AAC ACG ACA CCG GAT AA-3′ and 5′-GAG GTG CTT CAC TGT CAT TTC C-3′; Hey1, 5′-CGA CGA GAC CGA ATC AAT AAC-3′ and 5′-CAA ACT CCG ATA GTC CAT AGC C-3′; β-actin, 5′-CAC CAG GGC GTG ATG GT-3′ and 5′-CTC AAA CAT GAT CTG GGT CAT-3′. Expression levels were normalized to the mRNA expression of β-actin.

### CCK-8

Cells were seeded in 96-well plates. After indicated treatment, 10 μl of CCK-8 solution (Dojindo, Tokyo, Japan) was added to each well and incubated for 2 h at 37 °C. The optical density (OD) values were detected at 450 nm using a microplate reader.

### GSH measurement

GSH levels were measured by a GSH assay kit (Beyotime, Shanghai, China) following the manufacturer’s instructions. In brief, cells were deproteinated and the supernatant was processed to measure total GSH content with the 5, 5-dithio-bis-(2-nitrobenzoic acid), glutathione reductase and NADPH successively. The rate of change in absorbance was spectrophotometrically determined at 412 nm.

### ROS measurement

Cells were seeded in 6-well plates and treated with NAC (10 mM), GSH (20 mM) or Ebselen (10 μM) or respectively for 24 h. Subsequently, cells were incubated with 10 μM DCFH-DA (Beyotime, Shanghai, China) for 40 min and then harvested and washed with PBS. Finally, the intensity of DCFH-DA fluorescence was determined by flow cytometry at 480 nm (excitation) and 530 nm (emission).

### Cell cycle assay

Cell cycle assay was performed as previously described [20].Cells were seeded in 6-well plates and transfected with vectors. After that, cells were then cultured in DMEM with BSO (1 mM) for 12 h followed by NAC (10 mM) for another 24 h. Cells were harvested, washed with PBS and fixed in 70% cold ethanol (in PBS) at − 20 °C overnight. Following a wash with PBS, cells were incubated with 100 μg/ml RNase A (Solarbio) at 37 °C for 30 min and then stained with 50 μg/ml propidium iodide (Solarbio) in the darkness at 4 °C for 15 min. The cell cycle phase was determined by flow cytometry at 488 nm, and the relative ratios of the G1/G0, S and G2/M phases were analyzed by FlowJo.

### Annexin V- Propidium iodide (PI) assay

As previous reported [21], cells were seeded in 6-well plates and transfected with vectors. After that, cells were cultured in DMEM with BSO (2 mM) for 12 h followed by NAC (20 mM) for another 24 h. Cells were harvested, resuspended and fixed in 500 μl binding buffer. Subsequently, cells were incubated with 5 μl Annexin V-FITC (BD Biosciences, San Diego, CA) and 10 μl PI (BD Biosciences) for 10 min at 37 °C in the darkness. Cells were analyzed by flow cytometer within 2 h. Dot plots and histograms were analyzed by FlowJo.

### Wound healing assay

Wound healing assay was performed as previously described [22].Transfected cells were seeded in 6-well plates. When cells achieved 90% confluence, the middle of the culture was scraped with a sterile pipette tip (10 μl). The scratched cells were subsequently removed by washing with PBS and the wounds were viewed with a microscope and photographed. Cells were then cultured in DMEM with BSO (1 mM) for 6 h followed by NAC (10 mM) for another 12 h and images of wounds were captured. The scratch area was determined by Image J.

### Transwell invasion assay

Transwell invasion assay was performed as previously reported [23].Transwells (8.0 μm pore size, 24-well format, BD Biosciences) were coated with 50 μl 0.1% matrigel (BD Biosciences) and incubated at 37 °C for 4 h for gelling. Transfected cells were seeded in the upper chambers in 200 μl DMEM. The lower chambers were filled with 600 μl DMEM containing 10% fetal bovine serum. BSO (1 mM) was added to the upper chamber for 6 h exposure followed by NAC treatment (10 mM) for another 12 h. After that, cells from the top of the chamber membrane were gently removed and the invasion cells on the bottom of the membrane were fixed with 4% paraformaldehyde and stained with 0.1% crystal violet (Solarbio). Cells were photographed and counted in three randomly selected microscopic fields.

### Immunohistochemistry (IHC) assay

IHC analysis was performed as previously reported [24]. The tumor specimens were cut to about 4 μm sections and then embedded in paraffin for the immunohistochemistry assay. Tumor sections were stained with indicted antibodies. Images were obtained with Olympus microscope.

### Hematoxylin-eosin (HE) staining

HE staining was performed as previously reported [25].The tumor specimens were cut to about 4 μm sections and embedded in paraffin for hematoxylin-eosin staining. Images were obtained with Olympus microscope.

### TUNEL assay

TUNEL assay was performed as previously described [26]. The degree of apoptosis was evaluated by TdT-UTP nick end labeling (TUNEL) assay. The assays were performed with one-step TUNEL apoptosis assay kit (Beyotime Institute of Biotechnology) according to the manufacturer’s instructions. The FITC-labeled TUNEL-positive cells were imaged under a fluorescent microscope (Olympus, Japan). Cells with green fluorescence were defined as apoptotic cells. And images were analyzed by Image J.

### Animal studies

Xenograft model was established on BALB/C nude mouse (Animal Center of Tongji Medical College, Wuhan), 4–6 weeks old, weighing approximately 20-22 g. All studies involving animals were performed following the National Guides for the Care and Use of Laboratory Animals and approved by the Institutional Animal Care and Use Committee of Tongji Medical College, Huazhong University of Science and Technology.

A suspension of 1 × 10^6^ U87 cells (in 100 μl PBS) was subcutaneously injected into the right flank of each mouse. After the development of a palpable tumor (about 8 days, 5 mm in diameter), tumor size was measured with a caliper every 3 days and was reckoned by using the following formula: tumor volume = 1/2(width) ^2^ × length. Mice were divided into four groups: the control group (U87 cells and PBS injected) and the experimental groups (U87 cells and NAC/NAC + BSO/BSO treated), *n* = 3 per group. Animals were executed when tumor size reached the ethical end point.

### Statistics

Statistical analyses of the data were performed by t-test or ANOVA. Data were expressed as means ±SD from at least three independent experiments. *P* < 0.05 was considered statistically significant.

## Results

### NAC negatively modulates notch signaling activation in GBM

**Fig. 1:**
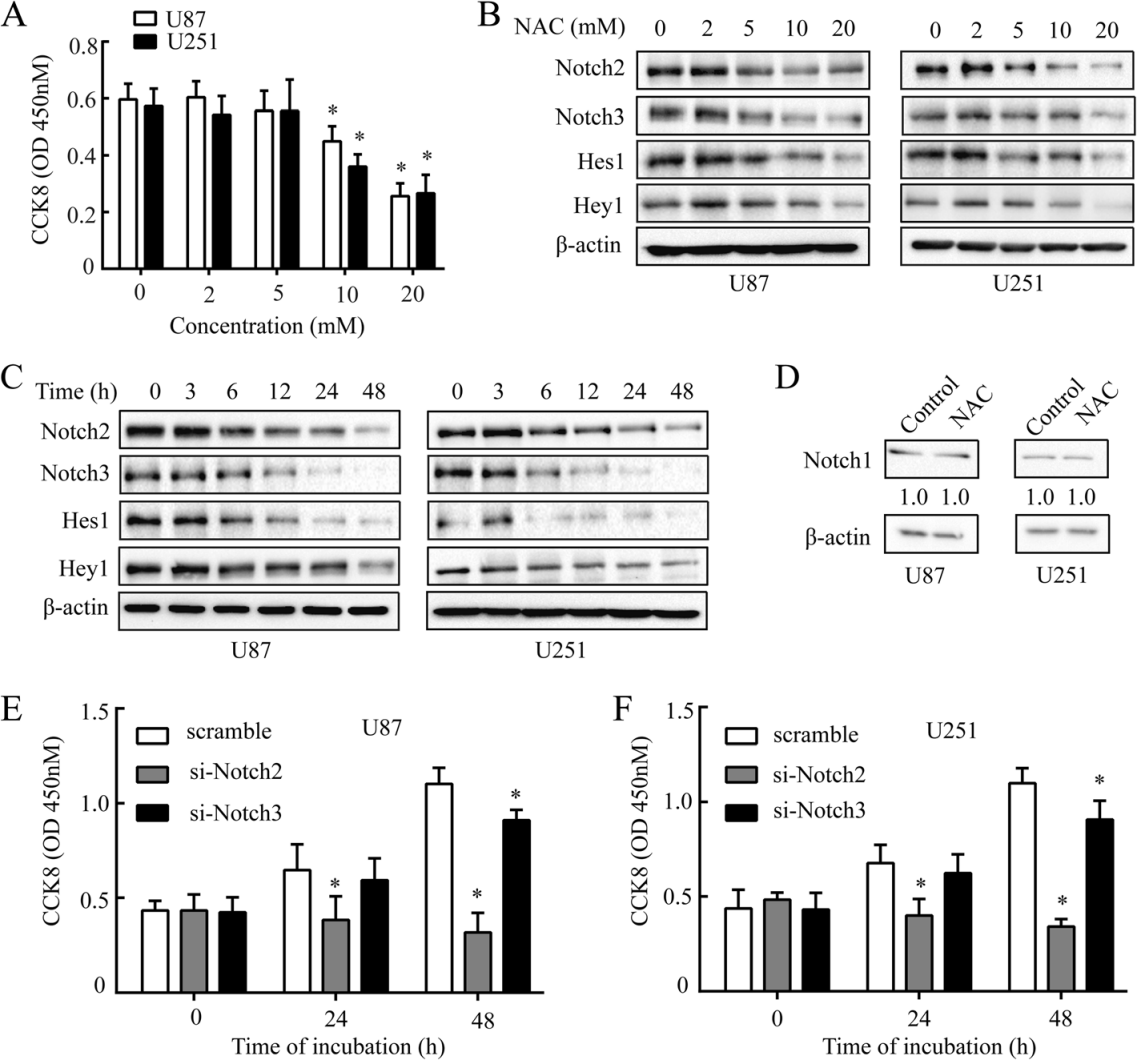
NAC decreases protein levels of Notch2, Notch3 signaling and restrains cell proliferation of GBM. **a**, Cell viability was analyzed by CCK8 at 450 nm. U87 and U251 cells were treated with NAC (2, 5, 10 or 20 mM) for 24 h. **b** and **c** The protein levels of Notch2, Notch3, Hes1 and Hey1 were analyzed by western blot. U87 and U251 cells were treated with NAC (2, 5, 10 or 20 mM) for 24 h (b) and NAC (10 mM) for 3, 6, 12, 24 or 48 h (**c**) respectively. **d** The protein levels of Notch1 were detected in U87 and U251 cells by western blot after NAC (10 mM) treatment for 24 h. β-actin was used as a loading control. **e** and **f** Cell viability was analyzed by CCK8 at 450 nm. U87 (**e**) and U251 (**f**) cells were transfected with Scramble, si-Notch2 or si-Notch3 (10 μM) respectively for 24 h and 48 h. Scramble served as a control. All data are presented as means ± SD of three independent experiments. * *P* < 0.05 compared with control group or Scramble group

NAC alone or in combination with other drugs has been widely used in prevention and treatment of many kinds of tumors [15–17]. However, the role of NAC in the therapy of GBM has not been clarified yet. In our study, NAC was found to inhibit cell viability of GBM cells at 10 mM and 20 mM effectively (Fig. 1a). Treatment of U87 and U251 cells with NAC resulted in a loss of Notch2 and Notch3 expression at different doses (0, 5, 10 and 20 mM) (Fig. 1b and Additional file 1:Figure S1A, S1B) and time points (0, 6, 12, 24 and 48 h) (Fig. 1c and Additional file 1:Figure S1C, S1D), but not Notch1 (Fig. 1d). Notch4 was undetectable in these cell lines [7].

Next, we wondered whether the down-regulation of Notch2 and Notch3 receptors caused by NAC would inhibit the downstream signaling, such as intracellular transactivation targets Hes1 and Hey1 [6]. The results suggested that U87 and U251 cells treated with NAC also led to dose (0, 5, 10 and 20 mM) and time (0, 6, 12, and 24 h) -dependent decreases in both protein (Fig.1b, 1c and Additional file 1:Figure S1E, S1F, S1G, S1H) and mRNA (Additional file 2:Figure S2A, S2B, S2C, S2D) levels of Hes1 and Hey1.

The dosage of NAC may determine whether NAC acts as a carcinogen or antitumor agent [27]. Since NAC had no effect on Notch2 at 2 mM or 5 mM but attenuated Notch2 expression at 10 and 20 mM, we used high dose of NAC at 10 mM in most of following studies. NAC was observed to take effect on GBM cells at 10 mM but not 5 mM, probably implying that Notch2 in GBM was relatively insensitive. Moreover, the dose of NAC at 10 mM is attainable in vivo, as similar concentrations of NAC have been intravenously administered in animals [28–30].

To further investigate the role of Notch2 and Notch3 in GBM cells, these two receptors were knocked down by their corresponding siRNA (Additional file 2:Figure S2E and S2F) and cell viability was analyzed. Both si-Notch2 and si-Notch3 (10 μM) impaired cell viability of U87 and U251 cells compared with scramble group as observed by NAC; however, si-Notch3 didn’t cause inhibition as significant as si-Notch2 did in both cell lines (Fig. 1e and f). It suggested that Notch2 may play a more predominant role than Notch3 in U87 and U251 cells. Since Notch2 showed much more correlation to the fate of GBM cells, we focused on the inhibitory effect of NAC on Notch2 in the following study.

### NAC regulates lysosomal degradation of Notch2

**Fig. 2:**
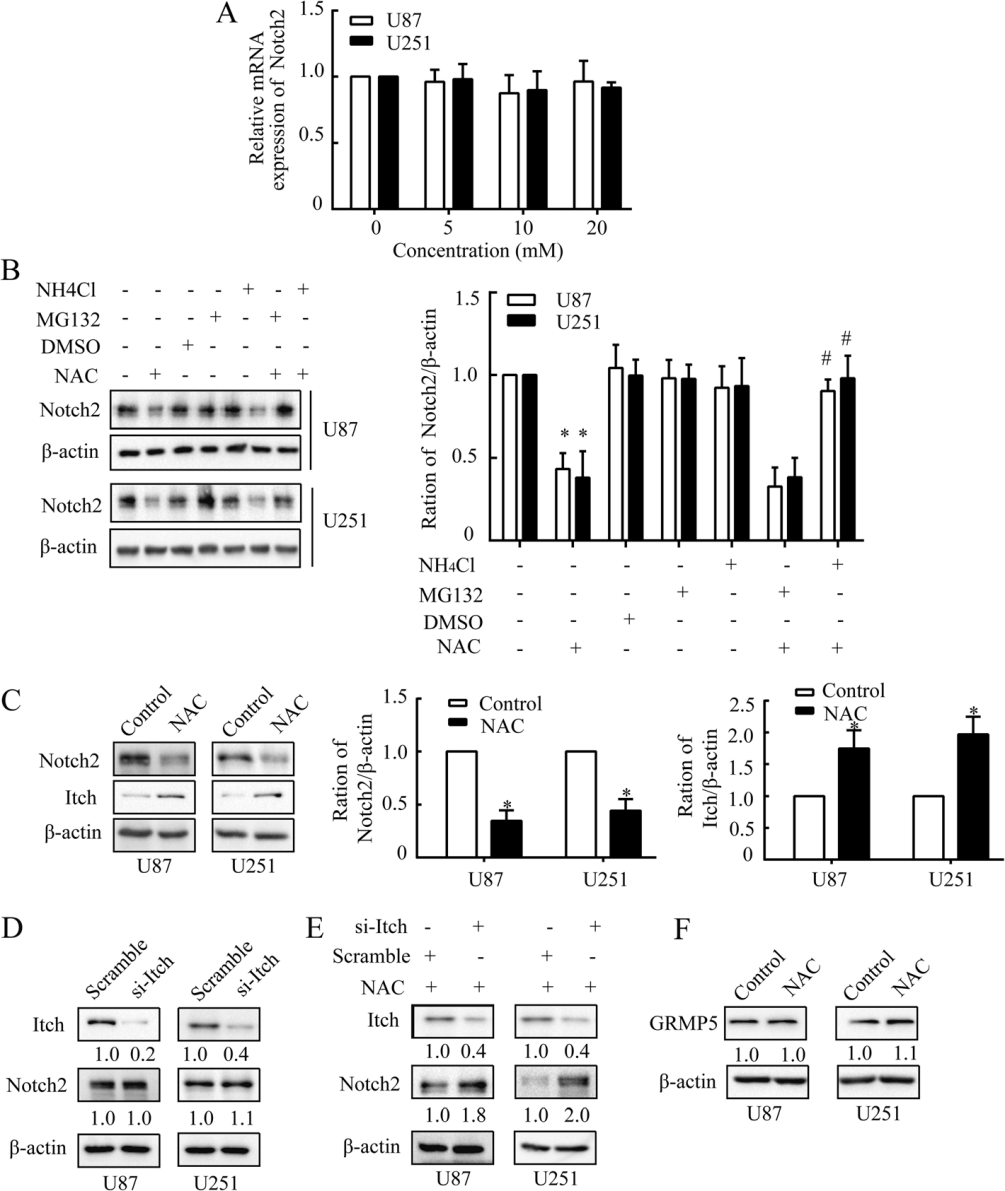
NAC diminishes Notch2 depending on lysosomal degradation. **a**, The mRNA analysis of Notch2 following dose-dependent treatment of NAC. U87 and U251 cells were treated with NAC (5, 10 or 20 mM) for 24 h. β-actin was used as a housekeeping gene. **b** The western blot analysis of Notch2 under proteasome (MG132, 10 μM) or lysosome (NH_4_Cl, 100 μM) inhibition and NAC (10 mM) treatment in U87 and U251 cells. **c** Notch2 and Itch were examined by western blot in U87 and U251 cells after NAC treatment (10 mM) for 24 h. **d** and **e** The western blot analysis of Itch and Notch2 after Itch silencing (**d**) or after NAC (10 mM) treatment in the presence of si-Itch (**e**) in U87 and U251 cells. **f** CRMP5 was analyzed by western blot in U87 and U251 cells after NAC treatment (10 mM) for 24 h. β-actin was used as a loading control. All data are presented as means ± SD of three independent experiments. * *P* < 0.05 compared with control group, # *P* < 0.05 compared with NAC group

To further explore the mechanism underlying NAC-mediated down-regulation of Notch2, the mRNA level of Notch2 was detected. The data implied that NAC did not interfere with Notch2 at mRNA level (Fig. 2a), indicating that the modulation events may occur at post-translational phase. Intracellular protein degradation may be achieved through proteasomal or lysosomal degradation pathways [31]. To testify these possibilities, NAC (10 mM) was applied to U87 and U251 cells following pre-treatment with MG132 (a proteasome inhibitor, 10 μM) or NH_4_Cl (a lysosome inhibitor, 100 μM), respectively. The results demonstrated that NH_4_Cl, but not MG132, could inhibit the decrease of Notch2 caused by NAC (Fig. 2b), suggesting that NAC-mediated Notch2 reduction was achieved by promoting its degradation through lysosome-dependent pathway.

Itch, an E3 ubiquitin ligase, might account for the attenuation of Notch receptors through lysosomal degradation [32]. So we tested whether Itch was involved in the suppression of Notch2 caused by NAC. The data showed that NAC (10 mM) up-regulated the expression level of Itch significantly (Fig. 2c), but Itch silencing (10 μM) had no impact on Notch2 protein in U87 and U251 cells (Fig. 2d), as also noted before [33]. To confirm whether the effect of NAC on Notch2 was controlled by Itch-dependent Notch degradation, NAC was applied in the presence of Itch silencing. Itch silencing could rescue the suppression of Notch2 by NAC (10 μM) (Fig. 2e), implying that Itch-dependent degradation was indeed involved in the suppression of Notch2 induced by NAC.

Given that collapsin response mediator protein 5 (CRMP5), a family member of five cytosolic proteins which are closely related to nervous system development, could protect Notch receptors from Itch-mediated lysosomal degradation in GBM [33], CRMP5 of GBM cells was detected after treatment of NAC, and no alternation of CRMP5 was observed in both cell lines (Fig. 2f). These data indicated that CRMP5 was not required for the inhibitory effect of NAC on Notch2 and there might be other molecules which worked together with Itch to mediate the degradation of Notch2 by NAC treatment.

### NAC decreases Notch2 through an antioxidant-independent pathway

**Fig. 3:**
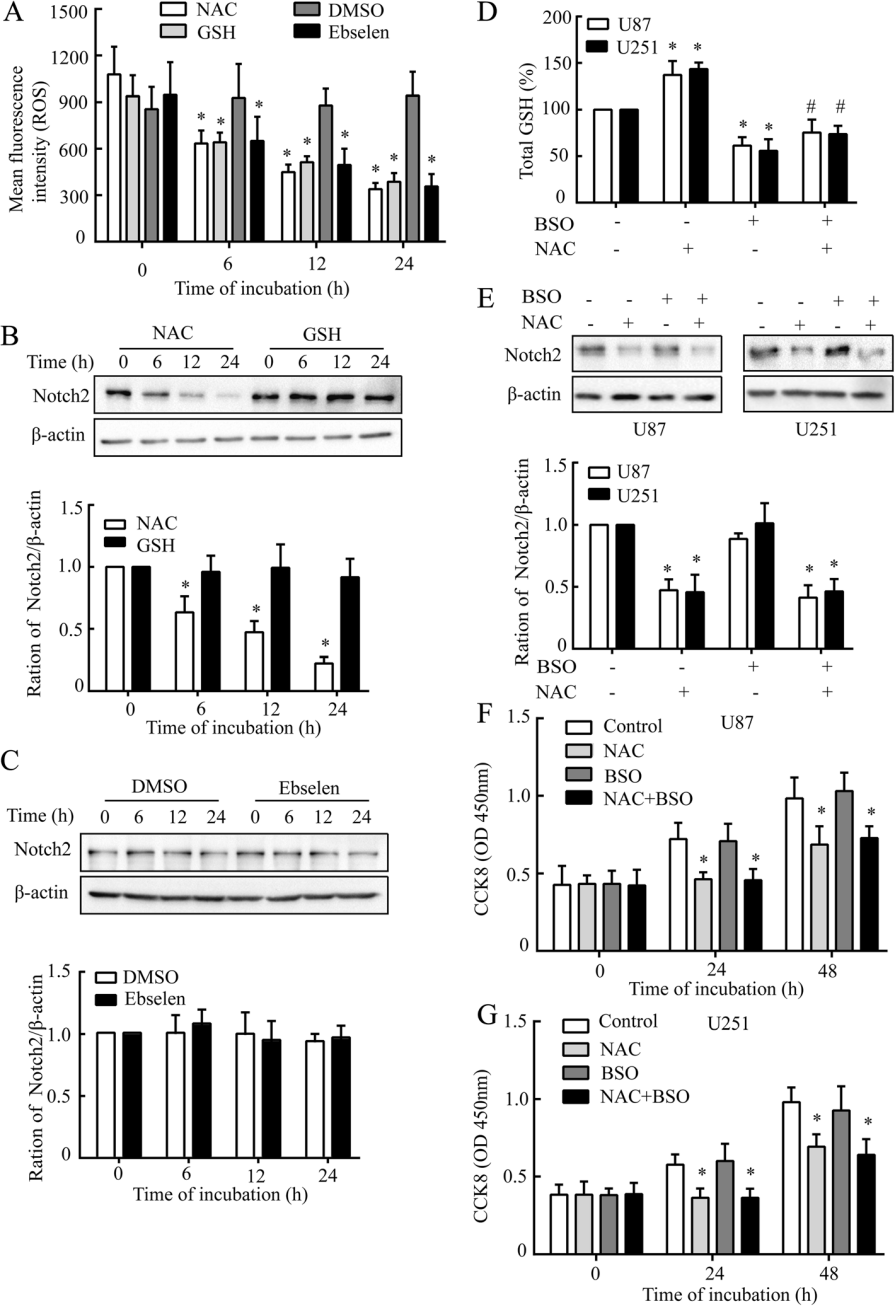
Independent of GSH in NAC induces Notch2 decrease. **a** The ROS levels were analyzed by flow cytometry using DCFH-DA (10 μM) following the treatments. U87 cells were treated with NAC (10 mM), GSH (20 mM) or Ebselen (10 μM) or for 6, 12 and 24 h respectively. **b** and **c** The western blot analysis of Notch2 under the treatment as **a** described. **d** Total cellular GSH was measured in U87 and U251 cells under pre-treatment of BSO (1 mM, 12 h), followed by NAC (10 mM, 24 h). **e** Effect of GSH depletion caused by BSO on Notch2 expression using western blot analysis. β-actin was used as a loading control. F and G, Cell viability was analyzed by CCK8 at 450 nm. U87 (**f**) and U251 (**g**) cells were treated as **d** described. All data are presented as means ± SD of three independent experiments. * *P* < 0.05 compared with control group, # *P* < 0.05 compared with NAC group

NAC was generally known to be an antioxidant [8]. To determine whether NAC-mediated Notch2 down-regulation is due to its conventional antioxidant activity, additional antioxidants, GSH and Ebselen, were used in this study. Intracellular ROS levels were detected firstly among these three antioxidants for comparison. NAC (10 mM), GSH (20 mM) and Ebselen (10 μM) all led to the decrease of ROS in U87 cells to almost a parallel degree (Fig. 3a); however, neither GSH nor Ebselen led to the reduction of Notch2 as NAC did (Fig. 3b and c).

To further confirm the antioxidant effect of NAC on Notch2, BSO, a glutathione-synthesis inhibitor [9], was applied with NAC in order to block the intracellular GSH produced by NAC. The results from GSH assays showed that NAC-mediated increase in intracellular level of GSH was effectively inhibited by BSO (1 mM) after 6 h of continuous exposure (Fig. 3d). However, the NAC-induced decrease of Notch2 was not altered by BSO (Fig. 3e). It demonstrated that NAC mediated the suppression of Notch2 through ROS-independent and GSH-independent pathways.

To investigate whether the action of NAC in growth arrest is related to its antioxidant property via intracellular GSH, we treated GBM cells with BSO in the presence of NAC and cell viability was analyzed. BSO itself had no effect on the growth of GBM cells (Fig. 3f and g). Despite the suppression of GSH accumulation (Fig. 3d), BSO did not affect the capacity of NAC to restrain the growth of GBM cells (Fig. 3f and g). The results above revealed that the effect of NAC on Notch2 and cell growth in GBM is independent of intracellular GSH.

### NAC attenuates proliferation of GBM cells via targeting Notch2

**Fig. 4:**
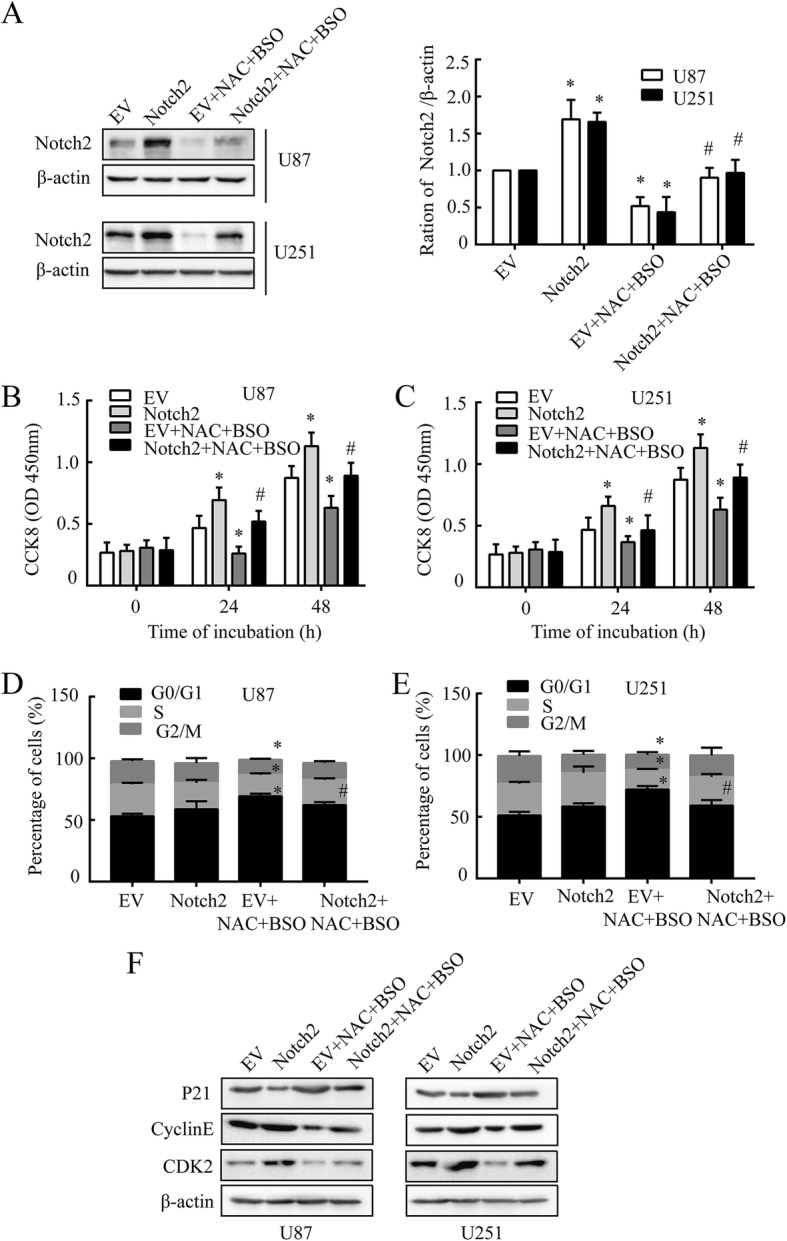
NAC attenuates proliferation of GBM cells through Notch2 signaling. **a**,Notch2 was analyzed by western blot. **b** and **c** Cell viability was analyzed by CCK8 at 450 nm. **d** and **e** The cell cycle analysis was measured by the percentage of cells in each phase in U87 and U251 cells. **f** The expression levels of P21, cyclin E and CDK2 in U87 and U251 cells. All cells were electroporated with pcDNA3.1-Notch2 or pcDNA3.1-EV, pcDNA3.1-EV served as a control, followed by BSO (1 mM, 12 h) and NAC (10 mM, 24 h) treatment. β-actin was used as a loading control. All data are presented as means ± SD of three independent experiments. * *P* < 0.05 compared with EV group, # *P* < 0.05 compared with EV + NAC + BSO group

To determine the role of Notch2 in NAC-induced growth arrest, pcDNA3.1-Notch2 and pcDNA3.1-EV (empty vector) were electroporated into U87 and U251 cells followed by NAC and BSO treatment. Compared with pcDNA3.1-EV, the pcDNA3.1-Notch2 resulted in a significant increase in Notch2 expression (Fig. 4a); and the pcDNA3.1-Notch2 rescued the growth inhibition caused by NAC (10 mM) at the same time (Fig. 4b and c). These data suggested that NAC-mediated suppression of cell growth in GBM cells was probably through Notch2 signaling.

Considering a defect of proliferation might be caused by an alteration of cell-cycle progression, the DNA content of GBM cells was measured. NAC (10 mM) treatment in the presence of BSO brought about G0/G1 arrest with a relatively increase in the G0/G1 phase population, accompanied by a corresponding reduction in the S and G2/M phase in U87 and U251 cells. G0/G1 arrest caused by co-treatment of NAC and BSO could be reserved by pcDNA3.1-Notch2 but not by pcDNA3.1-EV (Fig. 4d, e and Additional file 3:Figure S3A). These data further indicated that Notch2 was involved in the inhibition of NAC on GBM growth by inducing G0/G1 arrest, and this was independent of its antioxidation.

P21, a cyclin dependent kinase inhibitor, could lead to G0/G1 arrest by inhibiting the CDK2/cyclin E activities [34, 35]. It was reported that NAC induced G0/G1 arrest through the induction of p21 in hepatic stellate cells [9]. We then examined the cell cycle related check point proteins that controlled G1/S phase transition. Co-treatment of NAC (10 mM) and BSO (1 mM) gave rise to an increase in the expression of p21, but largely reduced CDK2 and cyclin E (Fig. 4f and Additional file 3:Figure S3B). Then we asked whether this effect was mediated by the decrease of Notch2 caused by NAC. The results illustrated that over-expression of Notch2 down-regulated the expression level of p21, but up-regulated CDK2 and cyclin E. Moreover, Notch2 over-expression could reserve the effect induced by NAC (Fig. 4f and Additional file 3:Figure S3B), demonstrating that NAC inhibited proliferation of GBM cells by altering the balance between p21 and CDK2/cyclin E via Notch2-depenent pathway. These data together proved that NAC might act as an inhibitor of Notch2 signaling and Notch2-dependent cell growth in GBM cells, and this was independent of its antioxidation.

### NAC induces apoptosis of GBM cells through Notch2

**Fig. 5:**
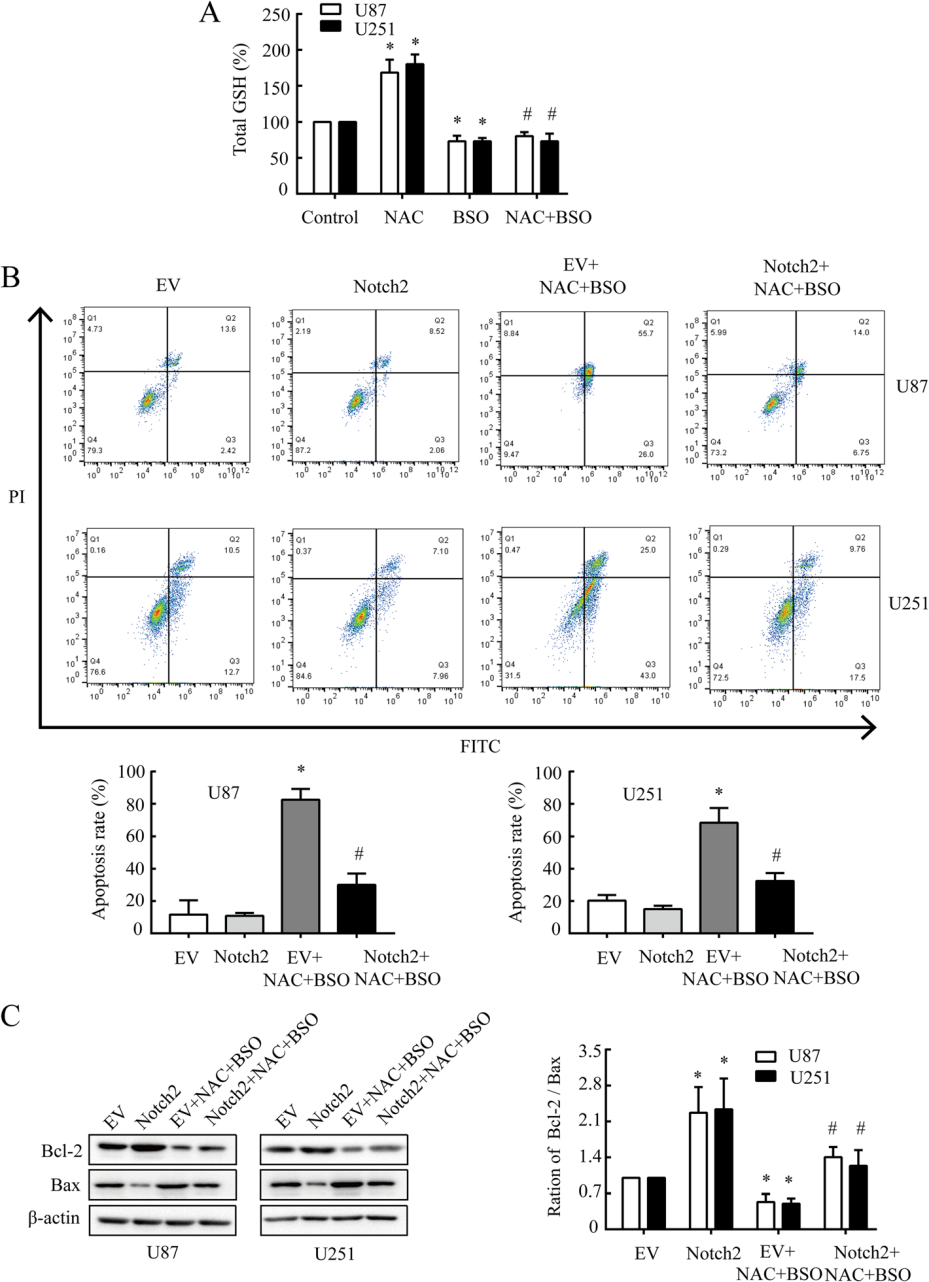
NAC induces apoptosis in GBM cells via targeting Notch2 signaling. **a** Total cellular GSH was measured in U87 and U251 cells under pre-treatment of BSO (2 mM, 12 h), followed by NAC (20 mM, 24 h). * *P* < 0.05 compared with control group, # P < 0.05 compared with NAC group. **b** Apoptosis rate was measured by flow cytometry assay.U87 and U251 cells were electroporated with pcDNA3.1-Notch2 or pcDNA3.1-EV, pcDNA3.1-EV served as a control, followed by BSO (2 mM, 12 h) and NAC (20 mM, 24 h) treatment. Q1: necrotic cells, Q2: late apoptotic cells; Q3: early phase apoptotic cells; Q4: normal cells. **c** The western blot analysis of Bcl-2 and Bax in U87 and U251 cells under the treatment as B described. β-actin was used as a loading control. All data are presented as means ± SD of three independent experiments. * P < 0.05 compared with EV group, # *P* < 0.05 compared with EV + NAC + BSO group

In previous trials, the phenomenon of cell death after NAC administration appeared at 20 mM. As NAC could induce p53-dependent apoptosis [36], we considered whether NAC would have effect on apoptosis with GSH depletion. The results of GSH measurement showed that BSO (2 mM) could block the GSH induced by NAC (20 mM) effectively (Fig. 5a). We further conducted experiments to assess the GSH levels in the presence of EV and Notch2. As shown in the additional file 4, over-expression of Notch2 could induce the increase of GSH in U87 and U251. Meanwhile, the NAC + BSO administration decreased the high level of GSH caused by Notch2 over-expression.

Flow cytometry analysis showed that the percentage of apoptosis cells increased significantly with NAC (20 mM) and BSO (2 mM) co-treatment in U87 and U251 cells (Fig. 5b). These results suggested that NAC facilitated cell apoptosis at a dose of 20 mM in a GSH-independent manner. Furthermore, NAC-induced apoptosis in GBM cells could be notably reversed by Notch2 overexpression.

Bax and Bcl-2 were reported to play a critical role in regulating cell death via apoptosis [37, 38]. In this study, the ratio of Bcl-2/Bax was observed decreased after NAC and BSO co-treatment, which could also be reversed by Notch2 overexpression (Fig. 5c). These data together suggested that NAC might lead to apoptosis at 20 mM in GBM cells via down-regulation of Notch2 through an antioxidant-independent pathway.

### NAC inhibits the migration and invasion of GBM cells through Notch2 pathway

**Fig. 6:**
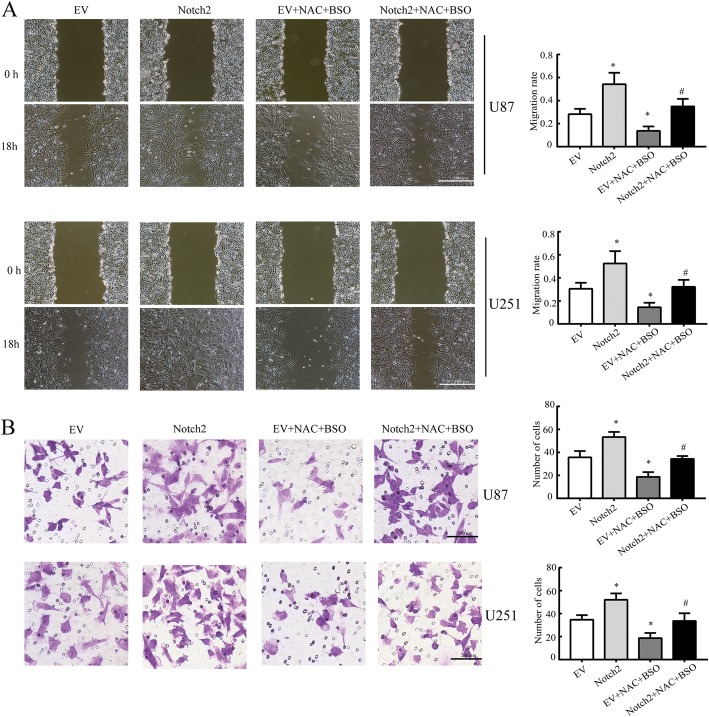
NAC inhibits migration and invasion of GBM cells by suppressing Notch2 pathway. **a** Migration rate was measured by wound healing assay. Scale bar: 500 μm. **b** Transwell invasion assays of U87 and U251 cells. U87 and U251 cells were electroporated with pcDNA3.1-Notch2 or pcDNA3.1-EV, pcDNA3.1-EV served as a control, followed by BSO (1 mM, 6 h) and NAC (10 mM, 12 h) treatment. Scale bar: 200 μm. All data are presented as means ± SD of three independent experiments. * *P* < 0.05 compared with EV group, # *P* < 0.05 compared with EV + NAC + BSO group

To examine whether NAC could inhibit the migration of GBM cells, wound healing assays were performed. NAC (10 mM) and BSO (1 mM) co-treatment inhibited the migration of U87 and U251 cells efficiently after scratched for 18 h (Fig. 6a). Activation of Notch2 pathway by pcDNA3.1-Notch2 transfection remarkably promoted the migration of GBM cells, and Notch2 overexpression rescued the inhibitory effect of NAC on migration (Fig. 6a), enhancing that NAC could suppress the migration of GBM cells via down-regulation of Notch2 in an antioxidant-independent manner.

Furthermore, to characterize the effect of NAC on the invasion of GBM cells, we conducted Matrigel invasion assays. Cells transfected with Notch2 showed higher invasion ability than those with EV. Compared with the EV group, significantly fewer U87 and U251 cells treated with NAC (10 mM) and BSO (1 mM) invaded the lower surface of the chamber (Fig. 6b). Importantly, the invasion ability of NAC in GBM cells was reserved by Notch2 overexpression (Fig. 6b). Taken together, these data indicated that the migration and invasion of GBM cells were attenuated by NAC through Notch2 signaling in a GSH-independent manner.

### NAC suppressed the growth of U87 cells in vivo

**Fig. 7:**
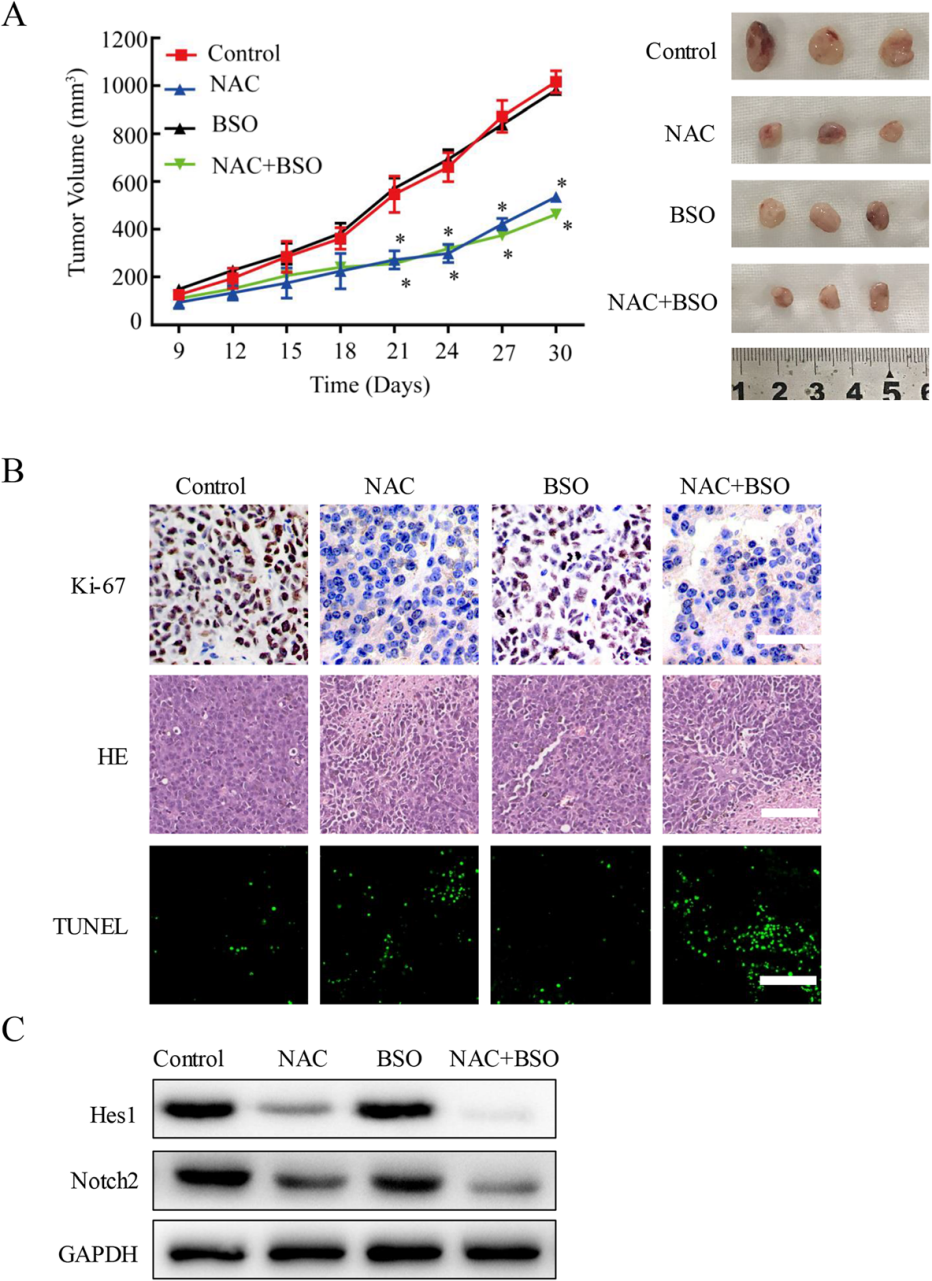
NAC induces apoptosis and inhibits proliferation of U87 cells in vivo. **a** Tumor growth curves for mice injected with PBS, NAC, BSO and NAC + BSO. The changes of tumor volumes are shown in the left panel and the dissected tumors in the right panel (*n* = 3, * *P* < 0.05 compared with PBS control group). **b** The representative histological examinations of the dissected tumors using Ki-67 staining, HE staining and TUNEL assay. Scale bar: 100 μm. **c** Notch2 and Hes1 were evaluated by western blot. All images shown are representative of at least three independent experiments

To further evaluate the role of NAC in vivo, xenograft model was established by subcutaneous injection of U87 cells in the flank of BALB/C nude mice. After tumors developed to about 400 mm^3^, we performed comparative efficacy studies by dividing mice into four groups (*n* = 3 per group) to minimize weight and tumor size differences among different groups. After that, mice were divided into four groups based on different treatments and injected via tail vein (PBS group, 200 μl;NAC group:100 mg/kg; BSO group:20 mg/kg;NAC + BSO group: BSO 20 mg/kg in the first day, NAC 100 mg/kg the next day). Tumor volumes of NAC alone and NAC + BSO groups were much smaller than those of PBS and BSO groups. The curve table and photographs demonstrated that NAC led to remarkable suppression of U87 tumors (Fig. 7a); NAC and NAC + BSO had significantly higher anticancer activity than PBS and BSO. These results were consistent with Ki-67 staining, HE staining and TUNEL assay, which showed that treatment with the NAC and NAC + BSO resulted in the lower level of proliferative capacity, higher level of necrotic lesions and higher apoptosis rate, respectively (Fig. 7b). As shown in Fig. 7c, Notch2 and Hes1 were evaluated by western blot. These data showed that, compared with PBS group, the expression of Notch2 and Hes1 increased in NAC and NAC + BSO groups, and not be influenced obviously in BSO group.

## Discussion

GBM is one of the most lethal brain tumors, and there is no significant curative effect observed in traditional cancer therapy [1, 39]. Cell proliferation of GBM has been extensively correlated to Notch signaling activation [3, 8, 40]. Notch1 and Notch2 are highly expressed in glioma cell lines as well as primary human gliomas [11, 41]. Overexpression of Notch1 could accelerate glioma cell proliferation and formation of neurosphere-forming stem cells [42]. The frequency and intensity of Notch2 were determined higher than those of Notch1 in GBM [43]. Notch2 has been known to drive embryonic brain tumor growth and genesis of GBM, playing a role in proliferation, differentiation and apoptosis [11, 12]. Based on our results, the inhibition of Notch2 caused by NAC may contribute to glioma therapy and its prognosis. The significance of Notch3 and Notch4 in GBM is not fully understood. Different from Notch2 [44], Notch3 has been implicated in choroid plexus tumors [45], and knockdown of Notch3 only slightly affect the viability of U87 cells [46],which is consistent with our observation. The absence of Notch4 receptor in our study is in accordance with a previous study which shows reduction of Notch4 [47], implying that Notch4 may act as a tumor suppressor gene in GBM.

The full-length Notch precursor (NFL) in Golgi complex was first cleaved by furinase (S1)-mediated proteolysis into extracellular (NEC) and intracellular (NIC) domains, which combined mutually to form mature Notch dimers. Then the activation of Notch signaling by its ligands would lead to sequential cleavages by tumor necrosis factor-a-converting enzyme (S2) and r-secretase (S3). NIC, the active form of Notch, then translocated into nucleus to promote the transcriptional activity of target genes [6]. Degradation of Notch protein may occur in the following two situations. First, NICD determines the potency of Notch signaling and proteolysis of NICD may account for attenuation of downstream targets. Second, NECD degradation also contribute to disease pathogenesis by preventing Notch activation from binding with ligands. Degradation of NICD and NECD could restrain Notch signaling and impair tumor growth. E3 ubiquitin ligases, such as Itch, Fbw7/Sel-10 and c-Cbl1, have the ability to catalyze ubiquitylation of Notch1 [48–51]. Furthermore, Itch/AIP4 has been shown to mediate ubiquitylation-dependent degradation of Notch1 through lysosome [12]. However, minor work has been described in the degradation of other Notch receptors (Notch 2–4). In our study, N2IC was found to be degradated in dependence on lysosome in the presence of NAC, thus we concluded that the elimination of Notch2 may require the lysosome system. N3EC and N3FL were covered in another research of cancer cells treated with NAC [31], and it demonstrated that the protein levels of N3IC and N3EC, but not N3FL, were decreased after NAC treatment, which indicated that NAC might target N3IC and N3EC in the non-covalent binding region directly or indirectly. Both CRMP5 and Itch knockdown would result in the degradation of Notch1 and Notch2 receptors through lysosome in GBM [22], so we took further examinations to explore the impact of NAC on CRMP5 and Itch. CRMP5 was not affected while Itch was increased, suggesting that NAC might not act on CRMP5 which would collaborate with Itch to mediate lysosome-dependent degradation of Notch2. The precise mechanism underlying NAC induced Notch2 degradation needs further investigation in future.

NAC has been known to be an antioxidant or a reducing agent [6]. In this study, GSH administration and GSH depletion by BSO showed no effect on the down-regulation of Notch2 caused by NAC, indicating that GSH level enhanced by NAC may not account for the Notch2 suppression. Meanwhile, the free radical scavenging activities of NAC were detected. Our results collectively indicated that inhibition of Notch2 signaling initiated by NAC was independent of its antioxidant property. Our findings might be strengthened by a previous study that NAC could decrease Notch3 independent of its antioxidant property [31]. An additional mechanism may be that the effect of NAC on Notch2 was due to its reducing activity by targeting its upstream signal-regulated molecules.

In accordance with vast majority of reports [16, 17, 27], application of NAC could attenuate cancer cell proliferation, migration and invasion. Moreover, NAC can regulate cell cycle progression, inhibiting the induction of cyclin D and DNA synthesis, which would lead to a G1 arrest in phorbol ester-induced NIH 3 T3 cells [13]. Hes1 and Hey1, known as direct downstream targets of Notch, belong to an extensive family of basic helix-loop-helix (bHLH) proteins and play a critical role in the regulation of cell cycle and apoptosis in various cancers [52, 53]. Both Hes1 and Hey1 are understood to promote G1-S transition by transcriptionally repressing p21 in a bHLH domain-dependent manner [52]. Our results showed that reduction of Hes1 and Hey1 caused by Notch2 inhibition may probably involve in the cell cycle arrest initiated by NAC.

The data reported here implied that Notch2 could play a predominant role in GBM multiplication, and the inhibition of Notch2 caused by NAC might contribute to glioma therapy.

## Conclusions

In summary, NAC could facilitate Notch2 degradation through lysosomal pathway in an antioxidant-independent manner. Meanwhile, NAC can attenuate Notch2 malignant signaling in GBM cells. Moreover, the notable ability of NAC to suppress cancer cell proliferation and tumor growth suggests that targeting Notch2 may serve as a promising strategy for developing future therapies of GBM, implying a novel application of NAC on GBM therapy.

## Additional files

Additional file 1: **Figure.** S1 NAC decreases protein levels of Notch2, Notch3 signaling. A, B, C and D, The relative expression levels of Notch2 and Notch3 were analyzed by western blot. U87 and U251 cells were treated with NAC (2, 5, 10 or 20 mM) for 24 h (A and B) or with NAC (10 mM) for 3, 6, 12, 24 or 48 h (C and D). E, F, G and H, The relative expression levels of Hes1 and Hey1 were analyzed by western blot. U87 and U251 cells were treated with NAC (2, 5, 10 or 20 mM) for 24 h (E and F) or with NAC (10 mM) for 3, 6, 12, 24 or 48 h (G and H). β-actin was used as a loading control. All data are presented as means ± SD of three independent experiments. * *P* < 0.05 compared with control group. (TIF 5225 kb)

Additional file 2: **Figure S2.** NAC decreases mRNA levels of Hes1 and Hey1. A and B, The mRNA analysis of Hes1 (A) and Hey1 (B) following dose-dependent treatment of NAC. Cells were treated with NAC (5, 10 or 20 mM) for 24 h. C and D, The mRNA analysis of Hes1 (C) and Hey1 (D) following time-dependent treatment of NAC. Cells were treated with NAC (10 mM) for 6, 12 or 24 h. β-actin was used as a housekeeping gene. E and F, The western blot analysis of Notch2, Notch3 using Scramble, si-Notch2 or si-Notch3 in U87 (E) and U251 (F) cells. β-actin was used as a loading control. All data are presented as means ± SD of three independent experiments. * *P* < 0.05 compared with control group or Scramble group. (TIF 6153 kb)

Additional file 3: **Figure S3.** NAC causes G1 arrest in GBM cells. A, The cell cycle analysis by measuring the percentage of cells in each phase using flow cytometry in U87 and U251 cells. B, The western blot analysis of P21, cyclin E and CDK2 in U87 and U251 cells. All cells were electroporated with pcDNA3.1-Notch2 or pcDNA3.1-EV, pcDNA3.1-EV served as a control, followed by BSO (1 mM, 12 h) and NAC (10 mM, 24 h) treatment. β-actin was used as a loading control. All data are presented as means ± SD of three independent experiments. * P < 0.05 compared with EV group, # *P* < 0.05 compared with EV + NAC + BSO group. (TIF 5721 kb)

Additional file 4: **Figure S4.** NAC and BSO decreased levels of total cellular GSH in GBM cells. A, Total cellular GSH was measured in U87 and U251 cells under pre-treatment of BSO (1 mM, 12 h), followed by NAC (10 mM, 24 h). B, Total cellular GSH was measured in U87 and U251 cells under pre-treatment of BSO (2 mM, 12 h), followed by NAC (20 mM, 24 h). All data are presented as means ± SD of three independent experiments. * *P* < 0.05 compared with EV group, # P < 0.05 compared with EV + NAC + BSO group. (TIF 5696 kb)

## Abbreviations

BSO: buthionine sulfoximine
GBM: Glioblastomas multiforme
GSH: glutathione
NAC: N-acetylcysteine
NECD: Notch extracellular domain
NICD: Notch intracellular domain
ROS: reactive oxygen species
